# Artificial intelligence for diagnosing bladder pathophysiology: An updated review and future prospects

**DOI:** 10.14440/bladder.2024.0054

**Published:** 2025-04-10

**Authors:** Chitaranjan Mahapatra

**Affiliations:** Institut Hospitalo-Universitaire, University of Bordeaux, Pessac 33600, France

**Keywords:** Bladder pathophysiology, Artificial intelligence, Machine learning

## Abstract

**Background:**

Bladder pathophysiology encompasses a wide array of disorders, including bladder cancer, interstitial cystitis, overactive and underactive bladder, and bladder outlet obstruction. It also involves conditions such as neurogenic bladder, bladder infections, trauma, and congenital anomalies. Each of these conditions presents unique challenges for diagnosis and treatment. Recent advancements in artificial intelligence (AI) have shown significant potential in revolutionizing diagnostic methodologies within this domain.

**Objective:**

This review provides an updated and comprehensive examination of the integration of AI into the diagnosis of bladder pathophysiology. It highlights key AI techniques, including machine learning and deep learning, and their applications in identifying and classifying bladder conditions. The review also assesses current AI-driven diagnostic tools, their accuracy, and clinical utility. Furthermore, it explores the challenges and limitations confronted in the implementation of AI technologies, such as data quality, interpretability, and integration into clinical workflows, among others. Finally, the paper discusses future directions and advancements, proposing pathways for enhancing AI applications in bladder pathophysiology diagnosis. This review aims to provide a valuable resource for clinicians, researchers, and technologists, fostering an in-depth understanding of AI’s roles and potential in transforming bladder disease diagnosis.

**Conclusion:**

While AI demonstrates considerable promise in enhancing the diagnosis of bladder pathophysiology, ongoing progresses in data quality, algorithm interpretability, and clinical integration are essential for maximizing its potential. The future of AI in bladder disease diagnosis holds great promise, with continued innovation and collaboration opening the possibility of more accurate, efficient, and personalized care for patients.

## 1. Introduction

The bladder is a muscular, hollow organ located in the pelvic region. It connects to the kidneys through the ureters and expels urine through the urethra. In males, it is closely linked to the prostate, while in females, it is adjacent to the uterus. Its primary function is to store urine until the body signals that it is time to void, at which point it contracts to push urine out. The bladder typically holds between 400 to 600 mL of urine.^1^ Pathophysiology of the bladder encompasses the functional changes that arise from various diseases and disorders. Disruptions in its normal physiology can lead to conditions such as bladder cancer, interstitial cystitis, or neurogenic bladder, which may involve structural, cellular, and molecular alterations.^2^ These changes can impact bladder control, storage capabilities, and overall urinary health, resulting in symptoms such as urgency, frequency, incontinence, or pain. For instance, bladder cancer is characterized by uncontrolled cell growth, whereas interstitial cystitis is hallmarked by chronic inflammation of the bladder walls.^3^,^4^ Understanding the mechanisms underlying these bladder disorders is crucial for developing effective treatments. Bladder pathophysiology, particularly conditions like overactive bladder (OAB), poses significant economic and social challenges, with billions spent annually on healthcare and lost productivity.^5^,^6^ In addition, these conditions can lead to heightened anxiety and depression among affected individuals. Early diagnosis of bladder disorders is essential for improving health outcomes and enhancing patients’ quality of life. Timely identification of conditions like bladder cancer or OAB allows for more efficacious management and intervention, reducing the risk of disease progression and treatment costs.^7^ For example, early-stage bladder cancer, if timely managed, has a better prognosis, leading to improved survival rates and less-aggressive treatment options.^8^ On the other hand, when bladder cancer is diagnosed at an advanced stage, treatment often involves radical cystectomy followed by urinary diversion. Although these interventions can be life-saving, they frequently result in significant changes in health-related quality of life (HRQoL). In one study,^9^ patients undergoing radical cystectomy with an ileal orthotopic neobladder showed an overall improvement in Global Health Status at 12 months, but outcomes varied by age.^9^ Receiver operating characteristic analysis identified the age of 70 years old as a threshold, beyond which radical cystectomy with orthotopic neobladder led to a significant decline in HRQoL. Based on these findings, ileal conduit diversion should be considered for patients older than 70 years to optimize post-operative quality of life. Patients may suffer from alterations in body image, social interactions, and daily activities due to the physical and psychological challenges associated with urinary diversion.^10^ In addition, the removal of the bladder and the necessity for continuous stoma care or a neobladder can adversely affect mental health and overall well-being.^11^ Addressing these issues requires a holistic, multidisciplinary approach that includes preoperative counseling, postoperative rehabilitation, and long-term support. Moreover, prompt recognition of urological issues can mitigate complications, relieve symptoms, and lessen the psychological burden associated with chronic bladder conditions.

Artificial intelligence (AI) refers to developing computer systems capable of performing tasks that typically require human intelligence, such as decision-making, pattern recognition, and problem-solving.^12^ In healthcare, AI has gained momentum as a transformative technology that can revolutionize diagnostics, treatment planning, patient monitoring, and even administrative processes.^13^ Machine learning (ML), a subset of AI, enables computers to learn from data patterns and make predictions, and is particularly useful in analyzing complex medical data.^14^ AI applications in healthcare are already making significant strides in fields such as radiology, oncology, and pathology.^15^ One specific instance is the use of AI-powered tools for breast cancer detection in radiology. Deep learning (DL) algorithms, such as those implemented in tools like Google Health’s AI system, have demonstrated high accuracy in analyzing mammograms. These systems can detect breast cancer with sensitivity comparable to or better than that of experienced radiologists while also reducing false positives and negatives. Studies have shown that integrating AI into mammographic screenings helps radiologists identify subtle abnormalities earlier, improving diagnostic accuracy and potentially leading to better patient outcomes.^16^ AI can help healthcare professionals make more informed decisions by analyzing a myriad of patient data. In addition, AI is employed in personalized medicine, helping to tailor treatments to the unique characteristics of each patient.^17^ As AI technology continues to evolve, its potential to improve diagnostic accuracy, reduce errors, and increase efficiency in the medical field is immense. AI has a growing significance in the field of bladder health, particularly in detecting and managing bladder-related issues. AI systems excel in image analysis and pattern recognition, which are vital for identifying abnormalities in bladder scans and histopathology.^18^ They can help delineate tumor boundaries and provide real-time feedback during procedures like cystoscopy.^19^ Furthermore, AI improves the accuracy of digital pathology, enabling pathologists to gain better insights into the histological features of bladder cancer. AI can also facilitate non-invasive urine testing by detecting biomarkers associated with bladder cancer and other urinary tract disorders.^20^ The ability of AI to enhance early diagnosis, minimizing bias in test interpretations, and create personalized treatment plans represents a promising advancement for patients with bladder pathophysiology.

Review papers are essential for elucidating pathophy-siological conditions by summarizing existing research and elucidating the mechanisms underlying diseases. They provide insights into functional changes in the body due to pathological states, enabling a better understanding of disease interactions. Moreover, these reviews often identify gaps in knowledge and propose areas for future exploration, contributing to our understanding of disease mechanisms and potential therapeutic targets. While there are significant advancements in AI applications within healthcare, there remains a gap in comprehensive review papers specifically connecting AI with bladder physiology. Addressing this gap is vital for a multidisciplinary understanding of how AI can improve our grasp of bladder pathophysiology and its treatment implications. By crafting a thorough review that investigates the relationship between AI and bladder physiology, this review aims to provide valuable insights for clinicians, pharmacologists, AI engineers, and physicists, fostering the development of innovative treatment strategies that can enhance patient outcomes and advance bladder health research. Moreover, this endeavor would lay the foundation for novel AI-driven approaches to bladder health, including predictive models for disease onset, personalized treatment plans, and innovative therapeutic techniques. By promoting cross-disciplinary collaboration and highlighting the untapped potential of AI in this field, a review on this topic would contribute significantly to both scientific knowledge and clinical practice, ensuring that AI’s capabilities are fully harnessed to improve bladder health outcomes.

## 2. Methods

A thorough and systematic search of the MEDLINE database was conducted through PubMed, focusing on peer-reviewed English-language articles.^21^ The search was designed to include both past and recent studies, ensuring comprehensive coverage of bladder pathophysiology and the evolving role of AI in the diagnosis of bladder-related conditions. The primary focus was on bladder disorders, such as bladder cancer, interstitial cystitis, OAB, underactive bladder, neurogenic bladder, bladder outlet obstruction (BOO), and other associated urological conditions. In terms of AI applications, studies that utilized advanced AI methodologies, particularly ML, DL, and neural network approaches – which have shown significant promise in enhancing diagnostic accuracy and prediction models – were included. The articles reviewed spanned diverse AI-driven approaches, from the development of predictive algorithms to clinical decision support systems tailored for urological diagnostics. Strict inclusion criteria were applied, with focus directed at original research articles, randomized and non-randomized clinical trials, observational cohort studies, case–control studies, and comprehensive review articles. By excluding non-English and duplicate articles, this review ensured the highest level of data integrity. In addition, the selection process emphasized the inclusion of high-impact and recently published studies to reflect the latest advancements in the field. To complement the primary search, supplementary references were also reviewed to identify any gaps or underexplored areas in AI’s application to bladder pathophysiology. This allowed for the development of a detailed conceptual framework, mapping AI’s role in improving the diagnostic workflow and accuracy for bladder disorders. In addition, this review also critically analyzed the challenges, such as potential biases, data quality issues, and integration limitations that could hinder the clinical adoption of AI tools in urology.

## 3. Bladder pathophysiology

### 3.1. BOO

BOO is a blockage at the bladder’s base or neck that limits urine flow. It is often caused by conditions such as benign prostate enlargement and benign prostatic hyperplasia in men, by pelvic organ prolapse in women, or by bladder stones, urethral strictures, bladder neck contracture, or tumors in both genders.^22^ BOO raises bladder pressure as the detrusor muscle strains to overcome resistance, leading to muscle thickening and reduced bladder elasticity. Over time, the bladder may lose its ability to empty fully, increasing the risk of urinary retention, infections, and bladder stones. In severe cases, damage of the bladder wall and kidneys may result, rendering early diagnosis crucial to avoid complications like hydronephrosis.

### 3.2. Neurogenic bladder

Neurogenic bladder results from disrupted nerve signals affecting bladder and sphincter coordination. It may arise from spinal injuries, multiple sclerosis, or diabetes and can lead to either overactive or underactive bladder symptoms.^23^ Overactive neurogenic bladder causes frequent, uncontrolled bladder contractions, while underactivity leads to retention. Detrusor-sphincter dyssynergia may occur, where the bladder contracts against a closed sphincter, raising pressure. Treatment aims to prevent complications and safeguard kidney health, often through behavior therapy, catheterization, or medication.

### 3.3. Interstitial cystitis or bladder pain syndrome (IC/BPS)

IC/BPS is a chronic condition hallmarked by bladder pain, urgency, and frequent urination without infection. Potential causes include bladder lining defects, neurogenic inflammation, and abnormal nerve sensitivity.^24^ Patients often report impaired quality of life due to constant pain and frequent urination. Treatment may involve medications, bladder instillations, physical therapy, and nerve stimulation.

### 3.4. Bladder cancer

Bladder cancer is a common urological malignancy, primarily affecting urothelial cells lining the bladder. It can manifest as non-muscle-invasive bladder cancer (NMIBC) or muscle-invasive bladder cancer (MIBC), with MIBC involving the bladder’s muscle tissue. Risk factors for bladder cancer include smoking, chronic bladder irritation (e.g., from infections or catheter use), and exposure to industrial chemicals such as aromatic amines and arsenic.^25^ Common symptoms include painless hematuria, urinary frequency, urgency, and dysuria. Treatment options are stage-dependent. For NMIBC, intravesical therapies such as Bacillus Calmette–Guérin immunotherapy or chemotherapy are standard, while MIBC typically requires radical cystectomy with urinary diversion, often in combination with neoadjuvant chemotherapy. Patients with advanced disease may undergo systemic chemotherapy or immunotherapy, such as immune checkpoint inhibitors targeting programmed cell death protein 1/programmed death-ligand 1.^26^

### 3.5. OAB

OAB leads to urgent, frequent urination, often with incontinence.^27^ It is caused by involuntary detrusor contractions and may be associated with nervous system conditions like Parkinson’s or stroke. Treatment includes lifestyle changes, pelvic floor exercises, medications, and, in resistant cases, Botox or nerve modulation.

### 3.6. Urinary incontinence

Urinary incontinence, or involuntary urine leakage, includes stress incontinence (caused by physical activities) and urge incontinence (caused by strong bladder contractions).^28^,^29^ It is commonly managed with behavioral therapies, pelvic exercises, medications, and surgical options for severe cases. Each bladder disorder has a unique pathophysiology, underscoring the need for individualized diagnosis and treatment strategies. Research advances improve our understanding of these conditions, leading to improved patient care.

## 4. Current diagnostic methods for bladder pathophysiology

Bladder pathophysiology presents unique challenges in both diagnosis and treatment. Accurate diagnosis is critical to tailoring treatment plans and improving patient outcomes. Conventionally, diagnosing bladder pathophysiology has relied on a combination of imaging techniques, urodynamic studies, and invasive procedures like cystoscopy. While these methods provide useful insights, they also have certain limitations that may impact diagnostic accuracy, patient comfort, and timeliness of treatment.

### 4.1. Cystoscopy

Cystoscopy is the primary diagnostic method for bladder conditions, particularly bladder cancer, by allowing for direct visualization of the bladder lining through a flexible camera inserted through the urethra.^30^ It is highly effective at detecting tumors, inflammation, and structural issues like diverticula or stones. However, as an invasive procedure, it can cause discomfort, particularly for those with high sensitivity or a narrow urethra, and carries risks such as infection, bleeding, and possible injury. While cystoscopy is highly sensitive for visible abnormalities, it may miss early or microscopic conditions, such as carcinoma *in situ*, especially in challenging areas such as the bladder and neck.^31^ It also lacks functional information on bladder dynamics, limiting its use in diagnosing conditions such as OAB or neurogenic bladder. New techniques, including narrow-band imaging and blue-light cystoscopy (BLC), enhance visualization using specific light wavelengths to highlight abnormal tissue better. BLC, in particular, has improved the detection of NMIBCs. However, these advancements still do not completely resolve the invasiveness and discomfort associated with traditional cystoscopy.

### 4.2. Urodynamic studies

Urodynamic studies are critical for assessing bladder and urethral function, especially in cases of urinary incontinence, OAB, and neurogenic bladder. These tests measure urine pressure and flow to evaluate how well the bladder and sphincter store and release urine. Key components include cystometry (which assesses bladder capacity and pressure), uroflowmetry (which measures urine flow rate), and pressure flow studies (which examine the relationship between bladder pressure and urinary flow).^32^ These tests help diagnose issues such as detrusor overactivity and BOO. However, urodynamic studies have several limitations. They are complex, require specialized equipment, and can cause discomfort due to catheter insertion. In addition, the invasive nature of these tests can alter normal bladder function. Moreover, they primarily identify functional issues rather than structural abnormalities, like tumors or strictures, that may impact symptoms.

### 4.3. Imaging techniques: Magnetic resonance imaging (MRI), computed tomography (CT), and ultrasound

Imaging techniques such as MRI, CT scans, and ultrasound are essential non-invasive methods for evaluating bladder structure and function.^33^ CT scans play a critical role in the staging of bladder cancer, particularly in evaluating the extent of local and regional spread, including lymph node involvement. However, its sensitivity in detecting metastatic lymph node involvement remains limited. According to recent findings, CT imaging often struggles to identify metastatic lymph nodes that are not significantly enlarged. Consequently, CT can produce false negatives in cases where metastatic lymph nodes are normal in size and false positives when benign lymph nodes are enlarged due to inflammation or other non-cancerous conditions.^34^ In one study, pre-operative CT scans showed that lymph nodes >15 mm were associated with a higher lymph node ratio (*p*=0.002) and an increased number of lymph nodes correlated with worse cancer-specific survival and overall survival (*p*=0.001 and *p*=0.002, respectively). A novel CT-based scoring system effectively stratified clinically node-positive patients into low- and high-risk groups for oncological outcomes (*p*<0.001).^35^ CT scans typically stage bladder cancer by assessing tumor spread, while MRI’s superior soft-tissue contrast helps visualize the bladder wall and adjacent structures.^36^ Ultrasound is often the first choice due to its accessibility, low cost, and ability to measure bladder volume and residual urine after voiding.^37^ It is particularly effective for detecting bladder stones, diverticula, and post-void residuals and can capture real-time bladder filling and ureteral jets. However, this imaging method has limitations. Ultrasound has a lower resolution than MRI or CT, making it less effective for detecting small lesions or early cancer. In addition, imaging quality may depend on the skill of the technician, and static imaging provides limited insight into bladder dynamics for conditions like OAB or stress urinary incontinence.

### 4.4. Emerging diagnostic approaches

Due to limitations in traditional diagnostics, there is increasing interest in non-invasive, more accurate tools, particularly those using advanced computing like AI. These technologies have the potential to transform bladder diagnostics by analyzing large datasets to identify patterns with improved accuracy. For instance, AI-driven analysis of urine biomarkers and cytological images can detect bladder cancer earlier, with higher sensitivity as compared to traditional methods.^38^ These tools can analyze molecular and imaging data that may be undetectable to the human eye, enabling earlier, more precise diagnosis. Non-invasive methods like photodynamic diagnostics (PDD) and autofluorescence are also gaining attention for enhancing bladder cancer detection.^39^ PDD uses a photosensitizing agent that accumulates in cancerous tissue, which is illuminated during cystoscopy to highlight abnormal areas. Autofluorescence distinguishes healthy from malignant tissue using natural fluorescence. These approaches have shown improved detection, particularly for early-stage cancers that are often missed in standard cystoscopy, reducing the need for repeated invasive procedures and enhancing patient comfort.

While traditional methods such as cystoscopy and imaging remain essential, these newer technologies could significantly improve diagnostic accuracy and patient outcomes by providing high-sensitivity, non-invasive options.

## 5. AI, machine learning, and neural network applications in medical diagnostics

**Figure 1:**
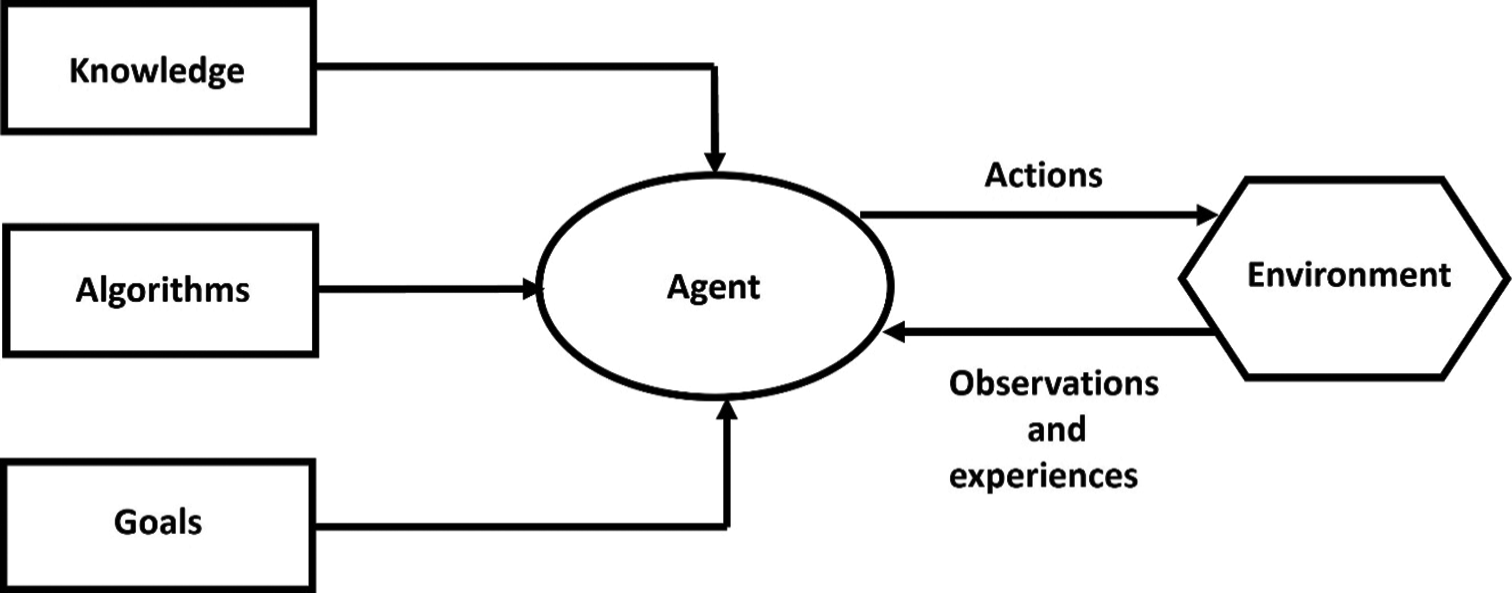
Basic schematic illustration of an artificial intelligence system. It represents connection with input nodes (knowledge, algorithm, goals), processing (agents’ decision-making), and output (actions and feedback).

AI comprises a diverse array of technologies designed to enable machines to perform tasks requiring intelligent, human-like responses. Core areas in AI include natural language processing (NLP), computer vision, and decision-making, all of which rely on advanced algorithms and extensive datasets.^40^ AI’s primary objectives include automating processes, making predictions, and performing complex cognitive functions, with its applications extending across various sectors such as healthcare, finance, and manufacturing.^41^ Recent advances in AI have produced systems capable of rapidly analyzing large data sets, identifying patterns, and making decisions with speed and accuracy that often surpass human capabilities. In AI systems, essential components include knowledge, algorithms, and goals, which collectively guide the system’s behavior. Knowledge offers foundational information and rules, algorithms enable data processing and decision-making, and goals specify desired outcomes. Operating within its environment, an AI system senses inputs, such as data from sensors or other stimuli, and determines actions based on these observations. This interaction includes a forward feedback loop, where actions affect the environment, and a backward feedback loop, providing information on the effectiveness of those actions, thereby refining future responses. A block diagram in Figure 1 represents this cycle with input nodes (knowledge, algorithm, goals), processing (agents’ decision-making), and output (actions and feedback).

AI, particularly in the form of ML and DL, plays a crucial role in the healthcare sector. Its applications include drug discovery, robotic surgery, nursing assistance, kidney disease forecasting, preliminary diagnosis, and research, among many others. Table 1 provides a detailed overview of AI’s contributions to various healthcare applications.

**Table 1:** Applications of artificial intelligence in healthcare

Healthcare applications	Detailed description
Drug discovery	Accelerate drug discovery and repurposing by leveraging extensive compound databases to evaluate chemical compounds and predict their effects and interactions.

Artificial intelligence-assisted robotic surgery	Equip surgical robots with real-time feedback capabilities to improve surgical accuracy and reduce the invasiveness of procedures.

Virtual nursing assistants	Monitor patients’ health conditions, provide answers to their inquiries, send reminders for medications, and alert healthcare professionals when needed.

Imaging analysis	Identify abnormalities, reduce radiation exposure, and improve image quality in computed tomography, magnetic resonance imaging, and ultrasound imaging methods.

Forecasting kidney disease	Evaluate the risk of chronic kidney disease or kidney failure by monitoring key indicators such as blood pressure, blood glucose levels, and demographic factors like age and family history.

Connected medical devices	Collect and analyze data from wearables, including smartwatches, that track key health indicators, physical activity, and vital statistics.

Preliminary diagnosis	Analyze symptoms, review medical history, and interpret test results to identify a probable diagnosis or compile a list of differential diagnoses for patients.

Prescription error recognition	Recognize and mitigate errors in prescribing and dispensing medications by checking for possible drug interactions, patient allergies, and accurate dosage calculations. This involves careful review of patient history, consistent monitoring for potential adverse reactions, and implementing protocols to ensure safe medication practices.

Researching and treating cancer	Analyze genomic data to identify mutations, classify tumor types, recommend personalized treatment options, and monitor patient responses throughout cancer treatment.

**Figure 2:**
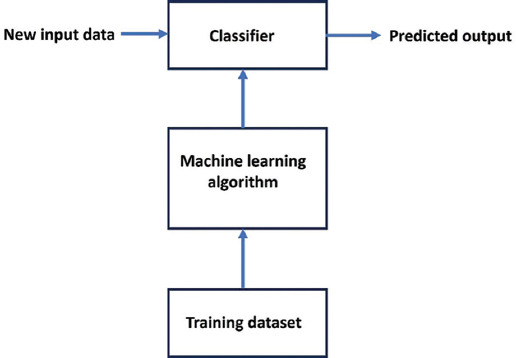
Block diagram of the machine learning process. The diagram shows the workflow from input data to output data.

Machine learning is a subset of AI focused on developing algorithms that enable systems to learn from data and improve their performance over time without explicit programming. ML algorithms analyze data, detect patterns, and make predictions by adjusting parameters based on feedback from their performance. These ML algorithms are categorized into supervised, unsupervised, and reinforcement learning, each being used for different types of tasks and data structures.^42^ ML is widely applied in areas such as recommendation systems, fraud detection, and medical diagnosis, helping systems adapt and evolve in response to new data. Figure 2 is a schematic representation of a simple ML process. This diagram illustrates the process of using ML for classification tasks, which could be applied to genomic analysis for personalized cancer treatment. The workflow begins with a training dataset that is fed into an ML algorithm. This algorithm uses the data to learn patterns, such as identifying genetic mutations or categorizing tumor types. After training, the algorithm outputs a classifier that can analyze new input data. When new input data (such as a patient’s genomic profile) are provided, the classifier processes this information and generates a predicted output, such as tailored treatment recommendations or predictions about patient responses. This process is essential for precision medicine, where insights from genomic data can guide individualized cancer therapies. DL is a specialized area within ML that employs multilayered neural networks to simulate the complex decision-making capabilities of the human brain.^43^ It excels in tasks such as classification and regression, handling vast amounts of unstructured data like images, text, and audio.

**Figure 3:**
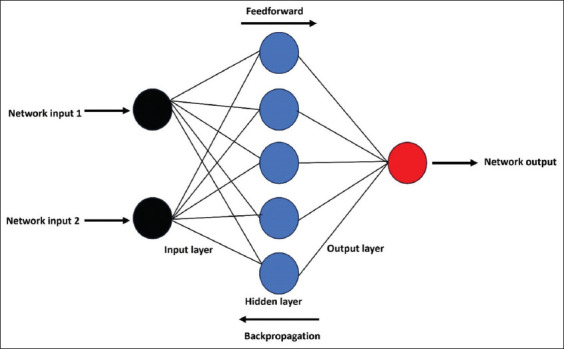
Block diagram of artificial neural network. It depicts a simple neural network comprising three layers: input, hidden, and output. Input signals (black nodes) are processed in the hidden layer (blue nodes) and combined in the output layer (red node) using linear weights to generate the final output.

Neural networks, inspired by the structure of the human brain, are a crucial aspect of DL within ML. They consist of layers of interconnected nodes (or “neurons”) that process input data in ways that mimic how neurons process signals. Through multiple layers and adjustments based on training data, neural networks can recognize complex patterns and perform sophisticated tasks, such as image and speech recognition.^44^ Using backpropagation and gradient descent, neural networks fine-tune their internal parameters to reduce error rates, becoming more accurate over time. These networks have led to significant advances in fields like autonomous vehicles and medical imaging. Figure 3 illustrates a simple neural network, which consists of three main layers: the input layer, the hidden layer, and the output layer. The input layer receives multiple input signals (black nodes), which are then processed through a set of weight-based functions in the hidden layer (blue nodes). The output layer (red node) combines the outputs of the hidden layer using another set of weights, known as linear weights, to produce a final output. This structure allows networks to approximate complex functions and patterns by leveraging localized responses in the hidden layer, making them suitable for tasks like function approximation, pattern recognition, and time-series prediction.

## 6. AI, machine learning, and neural network applications in medical diagnostics

AI is revolutionizing the diagnosis of bladder pathophysiology-related conditions by significantly enhancing accuracy, improving treatment predictions, and reducing the need for invasive procedures. Conditions such as bladder cancer, urinary incontinence, and interstitial cystitis pose considerable diagnostic challenges for healthcare professionals. Leveraging its capacity to analyze large volumes of complex data and identify patterns, AI is making substantial contributions to this field. By enhancing the precision of bladder cancer detection and streamlining the analysis of intricate urodynamic data, AI has the potential to significantly advance diagnostic methodologies. This discussion highlights several innovative AI strategies currently utilized in diagnosing bladder disorders, with an emphasis put on ML, DL, and applications in urodynamics and biomarker assessment.

### 6.1. Bladder cancer detection

Bladder cancer is one of the most common and potentially life-threatening urological diseases. Early and accurate diagnosis is crucial for improving patient outcomes. Traditional diagnostic methods, such as cystoscopy and biopsies, are often invasive and may lead to complications. AI-driven models, particularly ML and DL algorithms, have been developed to analyze medical imaging data, including MRI, CT scans, and cystoscopic images, for early bladder cancer detection. These AI models are capable of identifying tumors, segmenting bladder walls, and grading tumors based on the severity of malignancy. Supervised ML models have been developed using labeled datasets to diagnose bladder cancer. Supervised learning relies on historical data, where the model is trained using input-output pairs (e.g., clinical symptoms and diagnostic results), allowing the system to make predictions on new patient data. One common application of ML is the analysis of patient medical records and imaging data to predict the likelihood of bladder cancer. ML models have been used to predict bladder cancer recurrence and progression by analyzing patterns in histopathological data.^45^ These models evaluate the characteristics of bladder tissue samples, such as cell morphology and tumor grade, to assess the likelihood of recurrence after treatment. ML models are also increasingly used to predict the recurrence and progression of bladder cancer.^46^ One study^46^ developed a risk model for 1,062 patients with NMIBC treated with Bacillus Calmette–Guerin, providing recurrence (score: 0–16) and progression (score: 0–14) estimates. Compared to Sylvester tables, this model showed lower recurrence risks and lower progression probabilities in high-risk tumors, improving clinical decision-making. In addition, the ability of ML models to adapt and improve with the inclusion of new data offers an advantage over static prognostic models. Convolutional neural networks (CNNs), a subset of DL, have been particularly effective in analyzing bladder images, enhancing the accuracy of tumor detection by identifying subtle patterns that human radiologists might miss. This allows for earlier diagnosis, reducing the likelihood of cancer progression and the need for more invasive procedures.^47^,^48^ CNNs excel at identifying spatial hierarchies in imaging data, making them well-suited for detecting tumors or abnormalities within the bladder.^49^ In addition, CNNs have been used to enhance the accuracy of cystoscopic image analysis. Cystoscopy is a standard procedure for bladder cancer diagnosis, but it is often subjective and relies heavily on the experience of the clinician. By integrating CNNs into cystoscopic workflows, AI can automatically segment the bladder, detect lesions, and classify tumors, thereby improving diagnostic consistency and reducing the variability in results across different practitioners.^40^ Furthermore, DL models can assess not only tumor presence but also tumor stage, providing essential information for the development of treatment plans. In addition to improving detection, AI models can also predict the likelihood of tumor recurrence and response to chemotherapy. By analyzing large datasets, these models can personalize treatment plans, optimize patient outcomes, and minimize unnecessary treatments. AI’s predictive capabilities thus have the potential to revolutionize the management of bladder cancer, moving toward more patient-centered care.^47^

### 6.2. Urinary incontinence diagnosis

Urinary incontinence is another significant condition affecting the bladder, particularly among older adults and patients with neurogenic bladder disorders. Diagnosing urinary incontinence can be challenging due to the complexity of bladder function and the variability of symptoms. AI is proving to be an invaluable tool in this area, as it analyzes urodynamic data – measurements of bladder pressure, flow rates, and volume dynamics – along with patients’ medical histories. AI models can process and interpret urodynamic data faster than clinicians, identifying patterns that suggest the onset or progression of urinary incontinence. For instance, AI can analyze pressure-flow relationships in real-time during urodynamic studies, offering immediate insights into bladder performance. Predictive models are also being developed to identify patients at high risk of developing incontinence, thereby enabling earlier intervention and more effective management strategies.^50^ In a recent study,^51^ researchers developed and validated a prediction model for *de novo* stress urinary incontinence after vaginal pelvic organ prolapse surgery, achieving a concordance index of 0.73. The model outperformed expert predictions (area under the curve [AUC] 0.72 versus 0.62, *p*<0.001) and preoperative stress testing (AUC 0.72 vs. 0.54, *p*<0.001). An online calculator is available to aid clinical decision-making. By combining clinical data with AI-driven algorithms, clinicians can make more accurate diagnoses and suggest targeted treatments, such as physical therapy, medication, or surgery, based on individual patient profiles. This personalized approach enhances the effectiveness of interventions and improves the quality of life for patients suffering from incontinence. Cystoscopy is a critical tool in diagnosing urinary incontinence and determining appropriate treatments, such as deciding between a sling procedure and an artificial urinary sphincter. While AI is incrementally being integrated into functional urology, its application in directly analyzing sphincter function during cystoscopy is still in its nascent stages. Recent studies have highlighted the potential of AI in functional urology, particularly in evaluating lower urinary tract dysfunction. AI has been applied to enhancing diagnostic tools like dynamic MRI and urodynamics, which are essential for assessing sphincter function. For example, rapid diffusion tensor imaging has been explored for evaluating female urinary sphincter function by distinguishing between resting and contracting states.^52^ Although these AI-driven imaging methods are not yet standard practice, they hold promise for improving the accuracy of sphincter function assessment in the future. Moreover, AI-based recognition technologies have been developed to monitor urination activities, providing valuable insights for managing conditions such as neurogenic bladder. These systems utilize AI to automatically record and analyze urination patterns, aiding in the evaluation of urinary function and the prevention of dysfunction.^53^ While these advancements demonstrate significant progress, AI tools explicitly designed for analyzing sphincter function during cystoscopy remain limited. Continued research and technological innovations are expected to enhance AI’s role in this field, paving the way for more accurate diagnostics and personalized treatment strategies for urinary incontinence.

### 6.3. Interstitial cystitis detection

Interstitial cystitis, also known as painful bladder syndrome, is a chronic condition characterized by bladder pain and frequent urination. Diagnosing IC is notoriously difficult due to its symptoms overlapping with other bladder conditions, and it often requires invasive procedures like cystoscopy for confirmation. AI offers a promising alternative by analyzing a spectrum of data types – symptoms, biomarkers, and imaging results – to detect IC earlier and more accurately. ML algorithms have been designed to recognize patterns in patient-reported symptoms, such as pain intensity and frequency of urination, combined with laboratory results. A study,^54^ using ML algorithms, identified 87 differentially-expressed genes in IC/BPS, with placenta-specific 8 (*PLAC8*) (AUC: 0.887), S100 calcium-binding protein A8 (AUC: 0.818), and pro-platelet basic protein (AUC: 0.871) emerging as key diagnostic biomarkers. *PLAC8* overexpression promoted urothelial cell proliferation by inhibiting the protein kinase B/mammalian target of the rapamycin/phosphoinositide 3-kinase signaling pathway. These findings provide new insights into the immune landscape and potential diagnostic strategies for IC/BPS. AI systems are also capable of analyzing bladder biopsy images and urine samples to detect biomarkers associated with IC. This comprehensive data integration can lead to earlier diagnosis and more precise differentiation between IC and other conditions, like OAB, allowing for more targeted and effective treatments.^47^,^50^ In addition to improving diagnostic accuracy, AI can assist in monitoring treatment efficacy, allowing clinicians to adjust therapies based on the real-time analysis of patient responses. This approach minimizes trial-and-error in treatment and reduces the burden of unnecessary or ineffective interventions.

### 6.4. Predicting surgical outcomes

In the surgical treatment of bladder pathologies, particularly for invasive procedures like cystectomies (bladder removal), AI is emerging as a valuable tool for predicting patient outcomes. Surgical procedures carry inherent risks, and the success of surgeries, such as cystectomies, bladder augmentation, or Botox injections for OAB, is often dependent on numerous factors, including patient health, tumor stage, and bladder function. AI models can analyze preoperative data, such as imaging results, laboratory findings, and patient demographics, to predict the likelihood of surgical success. These models provide surgeons with valuable insights into which patients are most likely to benefit from specific surgical interventions. In a study,^55^ researchers developed a preoperative risk prediction tool for early mortality (within 90 days) after radical cystectomy, achieving an area under the receiver operating characteristic curve of 0.73. Key risk factors included the American Society of Anesthesiologists classification, congestive heart failure, age-adjusted Charlson co-morbidity index, and chronic pulmonary disease. This user-friendly tool enables clinicians to assess surgical risk using only preoperative data. By utilizing AI for surgical planning, clinicians can lower the risks associated with complex procedures, improving recovery time and overall patient satisfaction.^51^ AI’s predictive power extends to postoperative care as well. By monitoring patients’ recovery data and comparing them with historical datasets, AI can predict complications like infections, recurrence of bladder cancer, or the need for additional surgeries. This proactive approach allows for timely interventions, enhancing patient outcomes and reducing hospital readmissions.^56^

### 6.5. Urodynamic studies

Urodynamic studies provide valuable insights into the functional status of the bladder and urethra, particularly in diagnosing conditions like urinary incontinence, OAB, and neurogenic bladder. However, these studies often generate complex datasets that are difficult to interpret manually.^55^ AI techniques, especially ML models, can help in this domain by identifying patterns and correlations in urodynamic data that may go unnoticed by clinicians. For instance, AI algorithms have been used to analyze pressure-flow relationships and bladder compliance during urodynamic testing. In a study,^57^ a DL model using a short-time Fourier transform-based approach was developed to diagnose BOO and detrusor underactivity (DUA) from urodynamic data, achieving AUC scores of 0.945 and 0.929, respectively, in internal testing and 0.881 and 0.850, respectively, in external validation. This model demonstrated high accuracy and robustness in interpreting urodynamics. It has the potential to assist clinicians in diagnosing BOO and DUA in real-world clinical settings. By training ML models on large urodynamic datasets, researchers can create predictive models that assist in diagnosing functional bladder disorders with higher accuracy than traditional methods. AI can also help to stratify patients based on the severity of their symptoms, enabling more tailored and effective treatment strategies.^46^ AI-powered tools have also been applied to real-time analysis during urodynamic testing. These systems can provide immediate feedback on bladder performance, suggesting potential diagnoses based on the observed pressure and volume dynamics. Moreover, AI can help predict treatment outcomes for patients undergoing interventions such as Botox injections or bladder augmentation surgery by modeling how different treatments will affect bladder function.

### 6.6. Biomarker analysis for early diagnosis

AI is also being employed to analyze genetic, proteomic, and molecular biomarkers associated with bladder pathophysiology. Biomarkers are biological indicators that can be measured in blood, urine, or tissue samples and are increasingly used to diagnose and monitor diseases like bladder cancer. However, interpreting large-scale biomarker data requires advanced computational techniques, which is where AI excels. ML algorithms have been utilized to sift through vast amounts of genetic and proteomic data to identify biomarkers linked to bladder diseases. For instance, AI models can analyze urine samples for specific proteins or metabolites that are indicative of bladder cancer or IC. These biomarkers, when analyzed in combination with clinical data, can establish an early diagnosis before symptom manifestation or detection of abnormalities by traditional diagnostic methods.^47^ A previous study^58^ of urinary proteomic analysis identified 14 differentially-expressed proteins, including apolipoprotein A-I (apoA-I), as potential biomarkers for bladder cancer. Western blot analysis confirmed significantly elevated apoA-I levels in urine samples from bladder cancer patients compared to normal controls. These findings suggest that apoA-I and other identified proteins could serve as non-invasive biomarkers for bladder cancer diagnosis and surveillance. One promising area of research is the use of AI to analyze circulating tumor DNA (ctDNA) in blood samples. ctDNA is a fragment of genetic materials released by tumor cells into the bloodstream, and it acts as a non-invasive biomarker for detecting bladder cancer.^59^ AI algorithms can process this genetic information, identifying mutations and patterns associated with bladder cancer progression. This application of AI can not only assist in early cancer detection but also in monitoring patients for recurrence after treatment.^50^ From detecting bladder cancer and urinary incontinence to diagnosing IC and predicting surgical outcomes, AI-driven tools are revolutionizing how clinicians approach complex bladder conditions. By enhancing diagnostic accuracy, reducing the need for invasive procedures, and personalizing treatment plans, AI offers the potential to improve both the quality of care and patient outcomes in urological practice. As AI technologies continue to evolve, their integration into clinical workflows promises to reshape the future of bladder pathophysiology management.

## 7. Advancements in AI models and their relevance to diagnosing bladder pathophysiology

Advancements in AI, particularly with the emergence of large language models (LLMs) like ChatGPT, are revolutionizing clinical practice, research, and patient management.^60^ These tools have the potential to enhance diagnostic accuracy, support clinical decision-making, and improve communication between healthcare providers and patients. Exploring the integration of LLMs into bladder pathophysiology diagnosis and management reveals their capacity to transform workflows and patient outcomes in this specialized field.^61^ Sophisticated AI models like ChatGPT, developed using advanced NLP techniques, offer innovative solutions for healthcare applications. These tools can analyze and generate human-like text, making them valuable for tasks such as summarizing medical records, addressing clinical inquiries, and providing patient education – ChatGPT’s ability to generate contextually relevant texts is a promising resource in managing bladder pathophysiology. AI tools like ChatGPT enhance clinical decision-making by synthesizing extensive patient data, medical research, and diagnostic criteria. For example, ChatGPT can aid in diagnosing bladder cancer by analyzing patient histories, symptoms, and imaging results. By offering evidence-based recommendations, these tools streamline decision-making, reduce diagnostic errors, and expedite treatment timelines.^62^ AI can also help predict disease recurrence or progression by analyzing pathology reports, genetic data, and prior treatments, enabling the creation of personalized treatment plans for patients. Accurate diagnosis of bladder conditions – such as cancer, interstitial cystitis, or neurogenic bladder – often involves sophisticated imaging techniques like cystoscopy, CT scans, and MRI. While ChatGPT does not analyze images directly, its integration with imaging models can assist clinicians by interpreting radiological reports and identifying subtle abnormalities.^63^ In addition, AI models can process data from urodynamic studies to identify patterns indicative of specific pathophysiology and ultimately support clinicians in tailoring treatments. Effective communication is critical in managing bladder pathophysiology. AI models like ChatGPT can simplify complex medical information, making it accessible to patients with varying levels of health literacy. Patients diagnosed with conditions like bladder cancer or IC can use AI tools to receive clear, concise explanations about their diagnosis, treatment options, and post-treatment care. This enhances understanding, reduces anxiety, and promotes adherence to treatment plans, thereby improving clinical outcomes. In research, AI models significantly speed up the analysis of extensive scientific literature, helping identify emerging trends, biomarkers, and therapeutic targets in bladder pathophysiology. These tools assist researchers by summarizing findings, identifying gaps in knowledge, and generating hypotheses. AI can also support clinical trial design by analyzing datasets to uncover patient subgroups for targeted therapies, accelerating the discovery of effective treatments.

## 8. Challenges and limitations

While AI holds immense promise for improving the diagnosis and treatment of bladder pathophysiology, several challenges and limitations must be addressed to ensure its widespread and effective use in clinical practice. These challenges span technical, ethical, and organizational domains, all of which must be overcome to unlock the full potential of AI in healthcare.

### 8.1. Data quality and availability

A major obstacle in developing robust AI models is the availability of high-quality, annotated datasets. In bladder pathophysiology, comprehensive datasets that include imaging, genetic markers, and clinical records are still scarce. AI models require large, diverse, and well-annotated datasets to deliver accurate and reliable results. However, the collection of such datasets faces numerous challenges, including patient privacy concerns, inconsistent data collection methods across institutions, and limited access to rare condition cases like IC. In addition, the variability in data sources – ranging from different imaging equipment to varied clinical protocols – can significantly impact the performance and generalizability of AI models. As a result, models trained on a limited dataset may not perform well when deployed in different clinical environments.^50^,^54^ Moreover, ensuring data quality presents another challenge. Data can often be incomplete or contain errors, which can reduce the accuracy of AI algorithms. In many cases, manual review and data cleaning are necessary, which is time-consuming and resource-intensive. Therefore, efforts must be made to create large, standardized datasets with consistent and reliable data to improve AI model performance and generalizability in diagnosing bladder pathophysiology.

### 8.2. Ethical and legal considerations

The integration of AI into healthcare brings numerous benefits but also introduces critical ethical and legal challenges. A key concern is patient privacy, as AI systems rely heavily on extensive personal health data. Protecting this sensitive information is paramount, as breaches can expose private details, leading to misuse and a loss of trust. In addition, many AI systems operate as “black boxes,” with decision-making processes being not entirely transparent.^64^ This lack of interpretability complicates accountability. In cases of misdiagnosis or inappropriate treatment recommendations, it may be unclear whether responsibility lies with the clinician using the system or the algorithm’s developers. Another pressing issue is biases within AI systems. AI models trained on datasets that overrepresent certain demographic groups may fail to perform adequately for underrepresented populations. This inequity raises concerns about fairness and access to care, emphasizing the need to address biases during model development to ensure equitable outcomes for all patients.

#### 8.2.1. Data security in AI-powered healthcare

As AI and digital technologies become more embedded in healthcare, ensuring the security of patient information is critical. Breaches into healthcare data systems can lead to unauthorized access to personal health information, undermining patient confidence and exposing institutions to regulatory penalties.^65^ Effective strategies for safeguarding this data include:

(i) Encryption: Encrypting data both at rest and in transit using protocols like Advanced Encryption Standard and Transport Layer Security to prevent unauthorized access.
(ii) Access control: Employing role-based access controls to ensure only authorized personnel can access sensitive information, reducing internal security risks.
(iii) Anonymization and pseudonymization: Using anonymized or pseudonymized data during AI training to limit the impact of breaches by obscuring identifiable patient details.
(iv) Regular security assessments: Conducting routine audits and vulnerability assessments to identify and address weaknesses in AI systems and supporting infrastructure.
(v) Regulatory compliance: Adhering to standards such as the General Data Protection Regulation and the Health Insurance Portability and Accountability Act to protect patient data and mitigate legal risks.

#### 8.2.2. Lessons from data breaches

Incidents like the 2020 ransomware attack on Universal Health Services illustrate the consequences of inadequate cybersecurity. This breach compromised patient records and disrupted hospital operations, underscoring the importance of robust defenses. To prevent similar events, healthcare systems should integrate advanced security tools, such as real-time threat detection and mitigation technologies.

#### 8.2.3. Security challenges unique to AI

AI introduces specific security challenges that extend beyond traditional measures:

(i) Adversarial inputs: AI systems can be vulnerable to manipulated inputs designed to produce malicious outputs. Continuous training and validation are essential to mitigating these risks.
(ii) Algorithm transparency: Ensuring that AI algorithms are interpretable and auditable is vital for detecting and addressing potential security vulnerabilities.

While foundational measures like encryption and access control remain important, AI requires tailored solutions, such as securing model training environments and guarding against adversarial manipulation.^66^ Addressing these challenges demands collaboration among technology developers, healthcare providers, and policymakers to establish best practices for data security in AI. By learning from past breaches and implementing advanced safeguards, healthcare systems can leverage AI to improve clinical outcomes while maintaining the highest standards of data privacy and security.

### 8.3. Integration with healthcare systems

Seamless integration of AI technologies into existing healthcare systems remains a significant hurdle. Many healthcare organizations still rely on traditional systems that are not equipped to handle the computational and data storage needs of AI algorithms. Implementing AI-driven systems requires significant technical infrastructure, including advanced hardware, software, and cybersecurity measures. In addition, healthcare providers may need to undergo training to effectively use AI-based tools in their workflow. Regulatory challenges also impede the integration of AI into healthcare. Medical devices and diagnostic tools powered by AI are subject to stringent regulatory scrutiny to ensure they are safe and effective. AI systems must be validated and approved by regulatory bodies such as the Food and Drug Administration or equivalent authorities in other countries, which can be a lengthy and complex process. Without clear guidelines on how AI systems should be evaluated and integrated into clinical workflows, adoption may be slow and inconsistent across institutions.^42^

### 8.4. Acceptance by patients and clinicians

For AI to have a meaningful impact on healthcare, both patients and clinicians need to trust and accept its use. Clinicians are accustomed to traditional diagnostic methods, and many may be hesitant to rely on AI-driven systems, especially if they do not fully understand how the AI reaches its conclusions. Therefore, transparency in the AI’s decision-making process is vital. Clinicians need to be able to interpret the AI’s recommendations and have confidence that these suggestions are based on sound medical principles.^48^ On the patient side, the use of AI in healthcare may raise concerns about losing the human element of medical care. Patients may be uncomfortable with the idea of a machine making decisions about their health, particularly when it comes to sensitive issues, such as bladder diseases. Building trust in AI will require effective communication and education about the benefits of AI-driven care. Ensuring that AI systems complement rather than replace human clinicians may also help alleviate concerns among both patients and healthcare providers.

## 9. Future prospects, challenges, and limitations

The future of AI in diagnosing and managing bladder pathophysiology is filled with exciting potential, with various technological advancements expected to revolutionize the field. From the development of more sophisticated AI algorithms to their integration with personalized medicine and telemedicine platforms, the next phase of AI implementation in healthcare promises to significantly improve the diagnosis, treatment, and prevention of bladder-related disorders. Below are key areas where AI is expected to advance shortly.

### 9.1. Advances in AI algorithms

One of the most promising aspects of AI’s future in bladder pathophysiology is the continued advancement in AI algorithms. DL, ML, and NLP are evolving rapidly, offering the possibility of more accurate and robust diagnostic models. DL models, for instance, are particularly powerful in analyzing complex medical images and large datasets, which is essential for bladder cancer detection and monitoring overactive or neurogenic bladder conditions. NLP advancements can also assist in analyzing unstructured data, such as medical notes and patient records, providing a more holistic view of a patient’s health status. These improvements will make diagnostic models more precise, minimizing diagnostic errors and allowing for faster identification of bladder disorders. Enhanced accuracy means that AI systems can identify subtle patterns in medical data that may be missed by clinicians, leading to earlier and more personalized interventions.^49^ As AI algorithms become more sophisticated, they will integrate more seamlessly into clinical workflows, driving efficiency and improving patient outcomes.

### 9.2. Integration with personalized medicine

AI is uniquely positioned to advance personalized medicine, especially in the context of bladder pathophysiology. By analyzing genetic, proteomic, and clinical data, AI can help tailor treatments to individual patients, ensuring that therapeutic approaches are optimized for each person’s specific condition. For example, AI can evaluate the genetic markers of bladder cancer patients to predict which individuals are most likely to benefit from specific treatments, such as immunotherapy or chemotherapy. This approach holds the potential to revolutionize how bladder pathophysiology is managed. Personalized treatments not only lead to better patient outcomes but also reduce the likelihood of side effects and improve quality of life. AI’s ability to analyze vast amounts of data quickly and accurately renders it an ideal tool for implementing personalized medicine in clinical settings, providing more targeted and effective care to patients.^48^

### 9.3. AI in telemedicine

The integration of AI with telemedicine platforms is another area poised for growth. Telemedicine, which allows patients to consult with healthcare providers remotely, is especially beneficial for bladder health services. Many bladder conditions, such as urinary incontinence or OAB, can be monitored and managed through telemedicine, reducing the need for frequent in-person visits. AI-driven diagnostics can further enhance telemedicine by providing clinicians with tools to make accurate assessments remotely. For example, AI algorithms could analyze patient-reported symptoms, images, or home urine tests to detect early signs of bladder disease. This is particularly valuable for patients in underserved or remote areas, where access to specialized urologists may be limited.^51^ The combination of AI and telemedicine offers the potential to extend healthcare access to a broader population, enabling more timely diagnoses and treatment interventions.

### 9.4. Preventive healthcare

Preventive healthcare is another critical area where AI can make a significant impact. AI algorithms can identify individuals who are at high risk of developing bladder conditions, such as bladder cancer or OAB, based on their medical history, genetic data, or lifestyle factors. By flagging these high-risk individuals early, healthcare providers can implement preventive measures, such as lifestyle modifications, regular screenings, or early therapeutic interventions. This proactive approach has the potential to prevent the progression of bladder conditions, reducing the burden on both the healthcare system and the patients. For example, AI could help identify patients who might benefit from smoking cessation programs or dietary changes to reduce bladder cancer risks. Over time, AI-driven preventive healthcare strategies could improve public health outcomes and reduce the incidence of bladder diseases.^50^

The advancement of AI in bladder pathophysiology relies on overcoming existing challenges and limitations within the broader scope of healthcare. Future efforts must prioritize the development of robust, interpretable, and generalizable AI models, enabling their application across diverse clinical environments. A crucial step in this direction is fostering data sharing and collaboration among healthcare institutions, which would facilitate the creation of larger, more diverse datasets essential for training AI systems. This is particularly relevant for rare bladder conditions, where limited data currently impede progress in AI-based solutions. Addressing ethical and legal concerns is equally critical as AI becomes more integrated into medical practice. Key areas of focus include safeguarding patient privacy, mitigating algorithmic bias, and ensuring accountability. Transparent and explainable AI models are essential, allowing clinicians and patients to understand and trust the decisions generated by these systems. Research aimed at enhancing the interpretability of AI models will be pivotal in building this trust. In summary, the future of AI in diagnosing and managing bladder pathophysiology holds great promise. Advancements in algorithm design, personalized medicine, telemedicine, preventive care, and research are expected to revolutionize bladder health services. As these technologies continue to evolve, they are poised to significantly enhance the precision, accessibility, and overall quality of patient care, marking a transformative step forward in healthcare.

## 10. Conclusion

AI holds transformative potential in the diagnosis and management of bladder pathophysiology. Traditional approaches often encounter challenges such as limited diagnostic accuracy and invasive procedures, whereas AI offers solutions by enhancing precision and enabling personalized treatment strategies. However, significant obstacles remain, including ensuring high-quality data, addressing ethical considerations, and integrating AI into existing healthcare frameworks. Carefully addressing these factors is essential to ensuring the responsible and effective application of AI in clinical practice. As AI algorithms continue to advance, their impact on healthcare is expected to deepen. From improving diagnostic accuracy to reducing costs and optimizing patient outcomes, AI can revolutionize bladder health services. This progress is particularly evident with the advent of sophisticated models like ChatGPT, which enhance clinical decision-making, improve diagnostics, and facilitate effective patient communication. The future of AI in bladder pathophysiology is promising. With continued innovation, these technologies are poised to transform not only diagnostic processes but also the overall management of bladder conditions. AI’s integration into urology could make healthcare more efficient, accessible, and centered around patient needs. Overcoming challenges such as data privacy, model validation, and system integration will be key to realizing its full potential. Ultimately, AI tools like ChatGPT could become indispensable in diagnosing and managing bladder disorders, reshaping the landscape of bladder healthcare for the better.

## Data Availability

Not applicable.
